# The role of the combination of bone and fall related risk factors on short-term subsequent fracture risk and mortality

**DOI:** 10.1186/1471-2474-14-121

**Published:** 2013-04-04

**Authors:** Kirsten MB Huntjens, Tineke ACM van Geel, Svenhjalmar van Helden, Joop van den Bergh, Paul Willems, Bjorn Winkens, Piet P Geusens, Peter RG Brink

**Affiliations:** 1 Department of Trauma Surgery, Maastricht University Medical Centre, School for Public Health and Primary Care (CAPHRI), P. Debyelaan 25, P.O. Box 5800, Maastricht, 6202 AZ, The Netherlands; 2 Department of General Practice, Maastricht University/Caphri, Maastricht, the Netherlands; 3 Department of Trauma Surgery, Isala klinieken, Zwolle, The Netherlands; 4 Department of Internal Medicine, Viecuri Venlo, the Netherlands; 5 Department of Internal Medicine, Maastricht University Medical Centre, Maastricht, the Netherlands; 6 Biomedical Research Institute, University Hasselt, Hasselt, Belgium; 7 Department of Orthopaedic surgery, Maastricht University Medical Centre, Maastricht, the Netherlands; 8 Department of Methodology and Statistics, Maastricht University, Maastricht, the Netherlands; 9 Department of Internal Medicine, Maastricht University Medical Centre, Maastricht, the Netherlands

## Abstract

**Background:**

We analysed whether a combination of bone- and fall-related risk factors (RFs) in addition to a recent non-vertebral fracture (NVF) contributed to subsequent NVF risk and mortality during 2-years in patients who were offered fall and fracture prevention according to Dutch fracture- and fall-prevention guidelines.

**Methods:**

834 consecutive patients aged ≥50 years with a recent NVF who were included. We compared subgroups of patients according to the presence of bone RFs and/or fall RFs (group 1: only bone RFs; group 2: combination of bone and fall RFs; group 3: only fall RFs; group 4: no additional RFs). Univariable and multivariable Cox regression analyses were performed adjusted for age, sex and baseline fracture location (major or minor).

**Results:**

57 (6.8%) had a subsequent NVF and 29 (3.5%) died within 2-years. Univariable Cox regression analysis showed that patients with the combination of bone and fall RFs had a 99% higher risk in subsequent fracture risk compared to all others (Hazard Ratio (HR) 1.99; 95% Confidence Interval (CI) 1.18-3.36) Multivariable analyses this was borderline not significant (HR 1.70; 95% CI: 0.99-2.93). No significant differences in mortality were found between the groups.

**Conclusion:**

Evaluation of fall RFs contributes to identifying patients with bone RFs at highest immediate risk of subsequent NVF in spite of guideline-based treatment. It should be further studied whether earlier and immediate prevention following a NVF can decrease fracture risk in patients with a combination of bone and fall RFs.

## Background

For persons above fifty years of age, a history of fracture doubles the risk of a subsequent fracture [1]. This risk is highest immediately after the fracture, with a 5- to 25-fold increase of subsequent fracture risk within the first months and years [1-6]. In patients with a recent fracture, other bone-related clinical risk factors, low bone mineral density (BMD) and fall-related risk factors are often present [7]. These risk factors are independently related to fracture risk and are used in algorithms to calculate fracture risk, like FRAX® [8] and Garvan Fracture Risk calculator [9]. In addition, fall-related risk factors predict not only the risk of subsequent falls, but also of fractures. Therefore, these risk factors are sometimes integrated in fracture prediction algorithms [9].

Fracture risk reduction has only been shown with specific anti-osteoporosis medication such as bisphosphonates, denosumab, raloxifene and recombinant PTH [4,10-17]. Fall prevention strategies decrease the risk of falls, however in these studies prevention of fractures was not demonstrated [18].

In the field of post fracture care, a Fracture Liaison Service is one of the initiatives to integrate evaluation of bone- and fall-related risk factors in patients attending the hospital with a recent clinical fracture [19].

The aim of the Fracture Liaison Service is to evaluate bone- and fall-related risk factors, to initiate fall prevention programs, adequate calcium and vitamin D supplementation and specific anti-osteoporosis medication when needed in order to reduce subsequent falls, fractures and mortality [4,6,11,19-21].

In this study, patients with a recent clinical fracture were assessed at the FLS at Maastricht University Medical Center for bone- and fall related risk factors and we hypothesised that over a 2-year follow-up period the subsequent fracture risk and mortality would be highest in patients with a combination of bone- and fall-related risk factors, even though these patients received anti-osteoporosis treatment and/or fall prevention.

## Methods

### Study design

The Fracture Liaison Service is a collaboration between the department of surgery, orthopaedics and internal medicine (rheumatology and endocrinology) and is based on the consensus guideline osteoporosis of the Dutch Institute for Health Care Improvement (CBO) [20]. The Fracture Liaison Service is coordinated by a specialised and dedicated fracture nurse.

Between September 2004 and September 2006 all consecutive patients older than 50 years with a recent non-vertebral fracture, who entered the level one trauma centre in the south of the Netherlands were invited to participate. Patients with pathological or vertebral fractures or living outside the postal area were excluded. All patients were prospectively followed for two years. The hospital database was searched for radiographically confirmed first and subsequent NVF, fracture location and date of occurrence. All NVFs (baseline and subsequent) were categorised according to International Classification of Disease (ICD)-9 and then pooled into 2 groups: major (hip, pelvis, proximal humerus, proximal tibia, multiple ribs or distal femur fracture), and minor (all other) fractures [3]. All groups were mutually exclusive. First and subsequent fractures were classified according to the main fracture. The national obituary database was searched to investigate whether patients were deceased.

The study was approved by the medical ethical committee of the hospital (MEC 03–194).

### Measurements

All patients, who were able and agreed to evaluate their fracture risk assessment, were invited to attend the Fracture Liaison Service. Medical history, current and past medication use, living situation, conditions concerning the occurrence of the fracture, dietary calcium and vitamin D intake were assessed. Additionally, bone- and fall- related risk factors were systematically assessed, and bone mineral density was measured by dual energy X-ray absorptiometry (DXA, Hologic QDR 4500) at the lumbar spine and femoral neck. Based on criteria of the World Health Organisation osteoporosis was classified as T-score of ≤ −2.5, osteopenia as T-score between −1.0 and −2.5, and normal BMD as T-score of >−1.0.

According to the national osteoporosis guideline the following bone- and fall-related risk factors were evaluated: a history of clinical fracture after the age of 50 years, family history of hip fracture, low body weight (<60 kg), glucocorticoid use and immobility (<4 hours per day) [20]. Vertebral fractures were excluded from this study, since the exact date of occurrence is often unclear. Patients were categorised as having a bone-related risk factor if they had osteoporosis or at least one of the above mentioned risk factors.

According to the national guideline on fall prevention the following fall-related risk factors were evaluated: a previous falls in the last 12 months (the fall leading to the current fracture was excluded), the presence of Parkinson’s disease, current use of psycho-active medication, urinary incontinence (defined as involuntary loss of urine) and articular complaints. Additionally, the Groningen Activity Restriction Scale (GARS) was used to estimate the disability in activities of daily living (ADL) [21]. Patients were categorised as having a fall-related risk factor if at least one of the risk factors mentioned above was present or the GARS showed low ADL.

According to the Dutch guidelines on osteoporosis and fall prevention patients started with Calcium and Vitamin D or a bisphosphonate in the presence of osteoporosis.

For the analyses, patients were categorised into subgroups according to the presence, combination or absence of bone- and fall-related risk factors: (1) patients with only bone-related risk factors, (2) patients with combination of bone- and fall-related risk factors, (3) patients with only fall-related risk factors, and (4) patients without bone- or fall-related risk factors. The rationale behind these groups is that there is a known treatable risk factor in group 1 and 2, but not in group 3 because in fracture prevention only bone targeted therapy has shown to reduce fracture risk and not fall targeted therapies.

### Statistical analysis

Differences between the groups were analysed using the chi-square or Fisher’s exact test for categorical variables. ANOVA and independent samples t-test for numerical variables. Kaplan-Meier and multivariable Cox regression analyses were performed using subsequent fracture and mortality as dependent variables (events), adjusted for age, sex and baseline fracture location (major/minor). For subsequent fracture as dependent variable, follow-up time started at time of current fracture (time = 0) and was defined as time between current fracture and subsequent fracture (= event), death or end of 2-year follow-up period (= censored). For mortality, follow-up time was calculated as time between current fracture and death (= event) or end of 2-year follow-up period (= censored). Schoenfeld residuals were used to check the proportional hazards assumption and, if violated, time-dependent Cox regression was used. Linearity was checked for continuous variables and, if violated, centered quadratic terms were included. A two-sided p-value ≤ 0.05 was considered statistically significant. All analyses were performed using SPSS for Mac (version 18.0.0; SPSS Inc., Illinois, USA).

## Results

In total 834 patients with a NVF were included. Fifty-seven (6.8%) patients sustained a subsequent NVF and 29 (3.5%) died within two years.

Table 1 shows the patient characteristics for the total studied population, and for patients with (n=57) and without a subsequent non-vertebral fracture within 2 years (n=777). In total, 51.2% of patients had least one bone- and 60.4% had at least one fall-related risk factor. One in four patients had a previous clinical fracture at 50+ years, almost one in five had a low body weight (<60kg) or a family history with a previous hip fracture. The most common fall-related risk factors were articular complaints (31.3%), >1 fall in the preceding year (26.0%) and exposure to psychopharmaca (22.2%). Compared to patients without a subsequent fracture, patients with a subsequent fracture were significantly older (70.3 vs. 67.1 years), more patients had impaired mobility (10.5% vs. 3.9%) and a previous fracture after the age of 50 (38.6% vs. 25.2%), but less often had urinary incontinence (24.6% vs. 13.6%). Additionally, patients with a subsequent fracture had more often a combination of at least one bone-and one fall-related risk factor (group 2, 56.1% vs. 38.9%, p<0.01) compared to patients without a subsequent fracture.

**Table 1:** Comparison of characteristics of patients with and without a subsequent fracture

		**Total (n=834)**	**Subsequent fracture (n=57; 6.8%)**	**No subsequent fracture (n=777; 93.2%)**	**P-value**
**Age (SD)**		67.3 (10.4)	70.3 (11.1)	67.1 (10.3)	0.023
**Sex n (%)**					0.022
	Women	608 (72.9)	49 (86.0)	559 (71.9)	
	Men	226 (27.1)	8 (14.0)	218 (28.1)	
**Fracture location n (%)**					0.874
	Major	286 (34.3)	19 (33.3)	267 (32.0)	
	Minor	548 (65.7)	38 (67.7)	510 (68.0)	
***Bone RFs (%)***					
Fracture 50+ yrs		218 (26.1)	22 (38.6)	196 (25.2)	0.027
<60 kg		149 (17.9)	11 (19.3)	138 (17.8)	0.770
Positive family history		144 (17.3)	12 (21.1)	132 (17.0)	0.433
Immobility		36 (4.3)	6 (10.5)	30 (3.9)	0.017
On glucocorticoids		7 (0.8)	0 (0)	7 (0.9)	1.000
At least 1 bone RF		427 (51.2)	36 (63.2)	391 (50.3)	0.061
***Fall RFs (%)***					
>1 fall last year		217 (26.0)	21 (36.8)	196 (25.2)	0.054
On psychopharmaca		185 (22.2)	18 (31.6)	167 (21.5)	0.077
Low ADL (before fracture)		61 (7.3)	8 (14.0)	53 (6.8)	0.043
Articular complaints		261 (31.3)	17 (29.8)	244 (31.4)	0.804
Urinary incontinence		120 (14.4)	14 (24.6)	106 (13.6)	0.023
Parkinson’s disease		5 (0.6)	0 (0)	5 (0.6)	1.000
At least 1 of the fall RF		504 (60.4)	39 (68.4)	465 (59.8)	0.201

P-value refers to differences between patients with and without subsequent fracture.

Chi-square and Fisher’s exact tests were used for categorical variables and independent-samples t-tests for numerical variables.

Abbreviations: ADL: activity of daily living.

### Subsequent fracture risk

#### Comparison of subgroups

**Figure 1:**
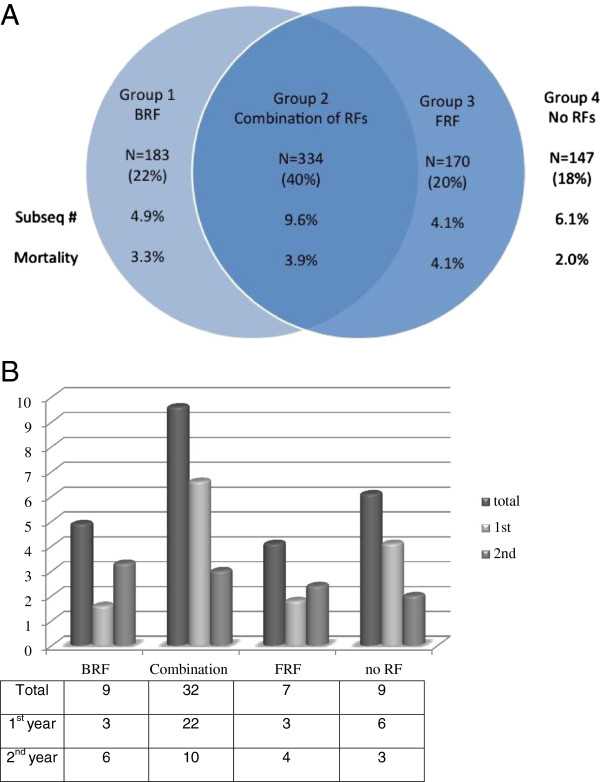
**All patients according to their pre-specified groups. A** Venn diagram. **B** Histogram.

**Figure 2:**
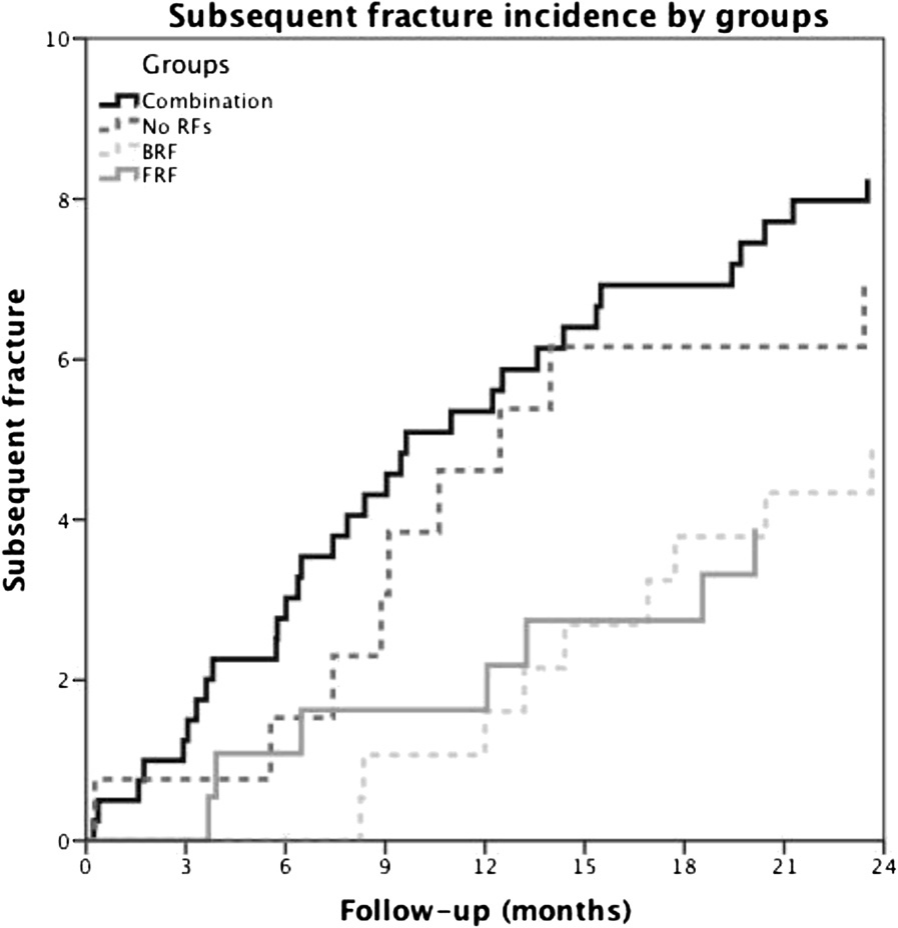
Multivariable Cox regression analyses stratified by groups.

Table 2 shows the results all patients according to the pre-specified groups. Patients with the combination of bone- and fall related risk factors (group 2, n = 334) were significantly older (70.0 years) compared with group 1 (n = 183, 65.6 years), group 3 (n = 170, 67.6 years) and group 4 (n = 147, 63.0 years), and were more often females (83.2%; 69.4% for group 1; 70.6% for group 3, and 56.5% for group 4). They had also sustained more often a major fracture (42.8%) at baseline compared to the other groups (group 1: 33.3%; group 3:27.6%; group 4: 23.8%, respectively). In absolute terms, no significant difference was found in absolute subsequent fracture risk between the groups (p=0.069) (Figures 1, 2 and Table 2).

**Table 2:** Comparison of all patients according to their pre-specified groups

		**Total n=834**	**Group 1 BRF n=183**	**Group 2 Combination of RFs n=334**	**Group 3 FRF n=170**	**Group 4 No RFs n=147**	**P value**
**Age (SD)**		67.3 (10.4)	65.6 (9.5)	70.0 (10.4)	67.6 (10.8)	63.0 (8.7)	
**Sex n (%)**							<0.001
	Women	608 (72.9)	127 (69.4)	278 (83.2)	120 (70.6)	83 (56.5)	
	Men	226 (27.1)	56 (30.6)	56 (16.8)	50 (29.4)	64 (43.5)	
**Fracture location n (%)**							<0.001
	Major	286 (34.3)	61 (33.3)	143 (42.8)	47 (27.6)	35 (23.8)	
	Minor	548 (65.7)	122 (66.7)	191 (57.2)	123 (72.4)	112 (76.2)	
**Subsequent fracture n (%)**	Total	57 (6.8)	9 (4.9)	32 (9.6)	7 (4.1)	9 (6.1)	0.069
	1^st^ year	34 (59.6)	3 (33.3)	22 (68.8)	3 (42.9)	6 (66.7)	
	2^nd^ year	23 (40.4)	6 (67.7)	10 (31.2)	4 (57.1)	3 (33.3)	
**Mortality n (%)**		29 (3.5)	6 (3.3)	13 (3.9)	7 (4.1)	3 (2.0)	0.728

Chi-square and Fisher’s exact tests were used for categorical variables and ANOVA F-tests for numerical variables.

#### Patients with a combination of bone- and fall-related risk factors versus all other patients

**Figure 3:**
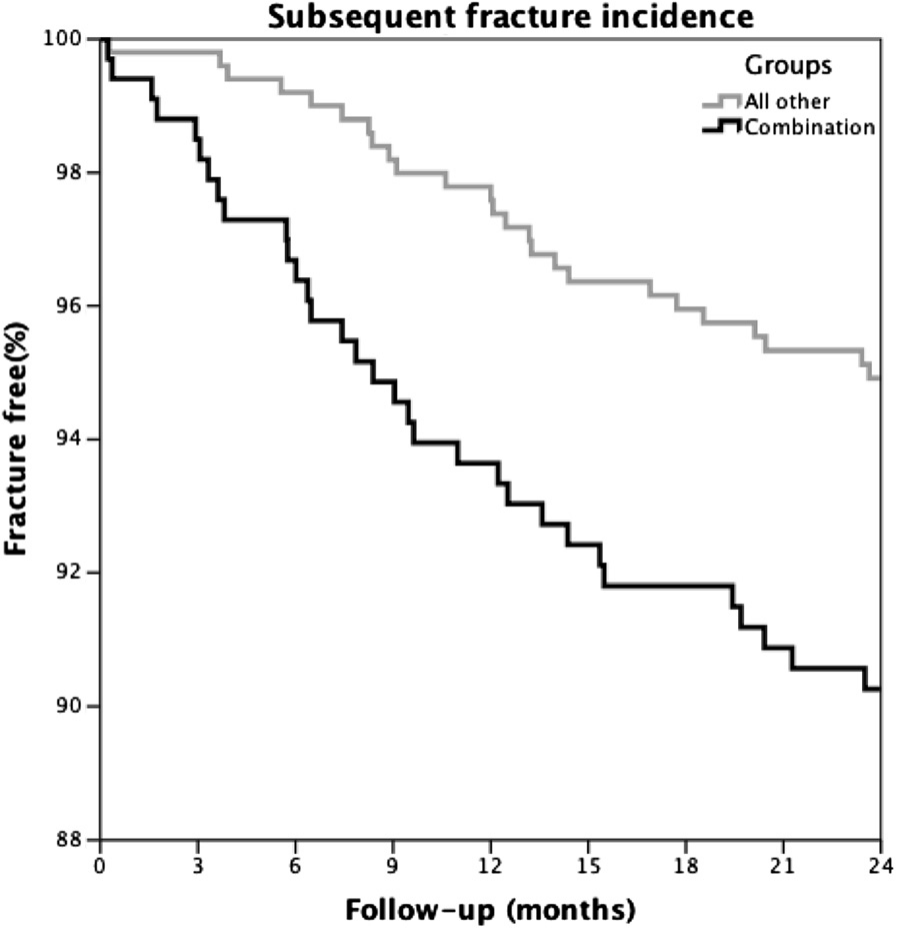
**Kaplan Maier.** Cumulative subsequent fracture incidence in patients with the combination of risk factors (group 2) compared with all other patients (group 1, 3 and 4).

Univariable Cox analysis showed that patients with a combination of risk factors had a significantly higher subsequent fracture risk than all other patients (HR: 1.99, 95% CI: 1.18-3.36 p=0.010) (Figure 3). However, after adjusting for age, sex and baseline fracture location (multivariable model) the subsequent fracture risk was not significantly higher (borderline) in the multivariable model (HR 1.70, 95% CI: 0.99-2.93; p=0.055).

#### Patients with a combination of risk factors compared to group 1 (only bone-related risk factors)

**Figure 4:**
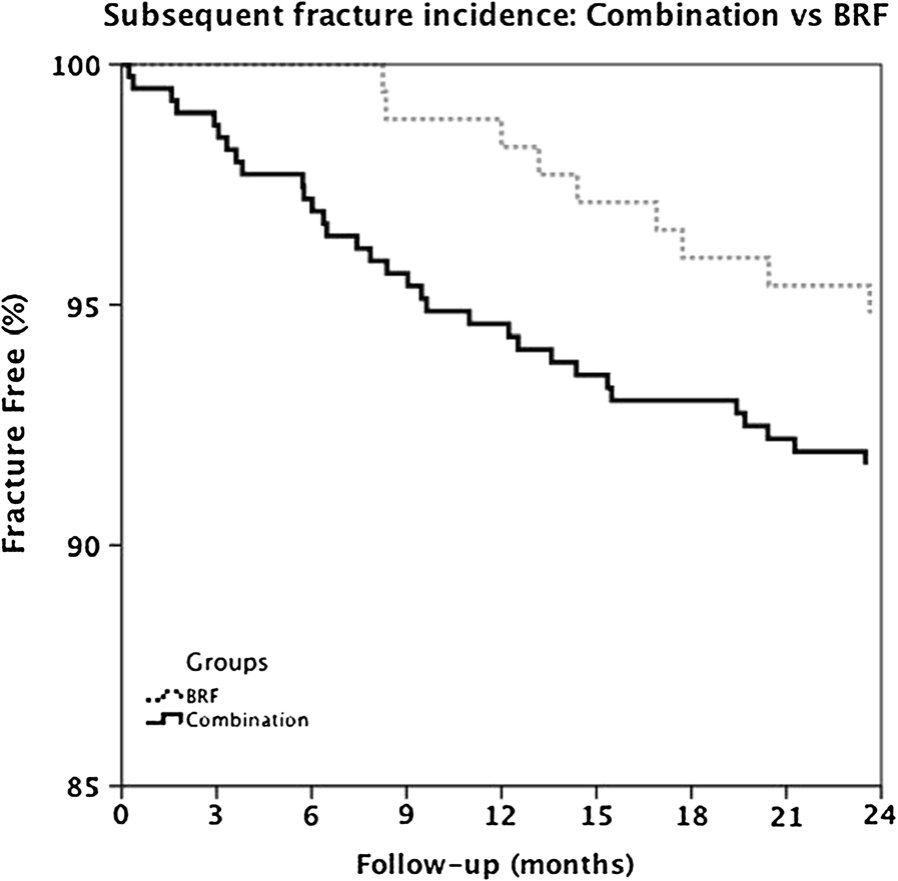
**Kaplan Maier curve.** Fracture free probability of the combination versus BRF, adjusted for age, sex and baseline fracture location.

Patients with the combination of risk factors did not have a significantly higher subsequent fracture risk than patients with only bone-related risk factors in univariable (HR 2.04; 95% CI: 0.97-4.27) or multivariable analyses (HR 1.67; 95% CI: 0.79-3.54). However, the plot of the Kaplan-Meier curves indicated that the subsequent fractures occurred immediately after the current fracture in the combination group, while this was not the case for the patients with only bone-related risk factors (Figure 4). This time-dependency was confirmed by the Schoenfeld residuals and the time-dependent Cox regression model (p = 0.024). However, the number of events is too low to draw reliable conclusions.

#### Patients with a combination of risk factors compared to group 3 (only fall-related risk factors)

In univariable analysis, patients with the combination of risk factors had a higher risk of subsequent fractures compared with patients with only fall-related risk factors (HR 2.41, 95% CI: 1.06-6.46). In the multivariable model, the subsequent fracture risk was no longer significantly higher (HR 2.05; 95% CI: 0.90-4.69).

#### Patients with a combination of risk factors compared to group 4 (no bone- or fall-related risk factors)

No significant differences were found between the groups on subsequent fracture risk within 2-years after univariable (HR 1.61; 95% CI: 0.77-3.38) and multivariable cox regression analyses (HR 1.16; 95% CI: 0.53-2.55).

#### Mortality

Within two years after baseline fracture 26 patients were deceased (3.5%). In univariable Cox regression analysis, no significant difference was found between patients with a combination of bone- and fall-related risk factors (group 2) and all other groups, as well as between group 2 and all groups separately. Due to the small number of deceased patients in the present study, no multivariable regression analyses were performed. However, this only reflects the very selected nature of our cohort, as the majority of patients were still alive within 2 years after their baseline fracture.

## Discussion

### Subsequent fracture incidence

The subsequent non-vertebral fracture risk in patients with a combination of bone- and fall-related risk factors was nearly double the risk of all other patients in univariable analysis (HR 1.99 (95% CI: 1.18-3.36). In multivariable analysis this tendency was also shown, but it did not reach significance (HR 1.70; 95% CI: 0.99-2.93). This is partly explained by the low event rate. There was a time-dependency when comparing patients with a combination of risk factors to patients with only bone-related risk factors (data not shown). This indicates that, in spite of Fracture Liaison Service assessment, patients with a combination of risk factors still had a high fracture risk at short term. Many studies have shown that the risk of subsequent fracture is highest immediately after a fracture, such as repeat vertebral, hip and non-vertebral fractures [3,6,22,23]. However, the event rate was lower in our research compared to other published articles. In a study among patients aged 60 years and over a relative risk of subsequent fracture incidence of 1.95 in women and 3.45 in men was found [3]. A subsequent fracture risk of 10.8% was found within 2-years after a fracture, and of 17.6% in patients of 50+ years who sustained a NVF after a NVF [4,5]. The two retrospective studies were performed according to intention-to-treat, and therefore, a difference in subsequent fracture rate and mortality could be found. Mortality is known to be increased after a fracture, especially after a hip fracture [24]. A recent study showed an increased risk of mortality especially within 5-years after the fracture [11]. In the described studies, [3-5,11,24] not all patients were treated at a Fracture Liaison Service. This might explain the lower subsequent fracture (6.8%) and mortality rate (3.5%) in our study compared with the studies mentioned above (due to a possible treatment effect). In addition, the minimum age of inclusion was different, and only patients who did attend the Fracture Liaison Service were included.

There was a time-dependency with regard to subsequent fracture risk in patients who had both bone- and fall-related risk factors with the highest risk immediately after the initial fracture. This might be caused by (new) falls and, since the bone is already vulnerable due to the initial fracture, subsequent fractures mostly occur in this subgroup short-term after the initial fracture.

Therefore, patients with a combination of bone- and fall-related risk factors, are definitely candidates for immediate fracture prevention combined with fall prevention.

However, of the patients without additional risk factors 6.1% had a subsequent fracture within 2-years of follow-up. Presumably other risk factors that are not captured by the bone- and fall- related risk assessment in this study play a role in the occurrence of subsequent fractures. On the other hand, all patients in this study had a recent fracture, which is by itself a major independent risk factor for subsequent fractures. As a consequence, this might imply that all patients with an initial NVF, whether or not additional risk factors are present, deserve subsequent fracture prevention regardless of other risk factors. This is also proposed in the UK guideline [25].

### Mortality

No difference was found between the groups for mortality rate. This could be the result of the low mortality rate (n=29) in this study in combination with the relatively short follow-up period (two years). A study with a longer follow-up period in a larger population is necessary to further study these findings. Treatment of osteoporosis with bisphosphonates might reduce mortality rate. The mechanism by which this operates remains unclear, but its effect on subsequent fracture risk or on extra skeletal sites might be two possibilities [11,26,27]. Integrating guidelines on prevention of falls, fractures and identification and treatment of osteoporosis is recommended to optimise post fracture care. However, integrating these services is difficult and still has to be achieved in many centers [28].

### Strengths and limitations

The strength of this study is that all consecutive patients with a recent non-vertebral fracture who attended the Fracture Liaison Service were included in the analyses. Furthermore, a dedicated fracture nurse performed the assessments of well-defined clinical, bone- and fall-related risk factors according to the national Osteoporosis and Fall Prevention Guidelines [20,21].

A limitation of this study is that there are no data on prescription and adherence of the proposed osteoporosis treatment and fall prevention. Persistence might be low, as has been shown in a recent publication for oral anti-osteoporosis medications in the Netherlands [29]. Due to the low mortality rate, no in depth analysis of the role of risk factors could be performed. However, this could also be a strength, since one of the effects of the Fracture Liaison Service might be mortality risk reduction.

## Conclusions

In patients with a recent non-vertebral fracture who had a combination of bone- and fall-related risk factors, the risk of subsequent fractures was almost doubled compared with the other patients. These patients also had a higher subsequent fracture risk immediately after the initial fracture compared with patients in whom only bone-related risk factors were present.

Therefore, not only bone- but also fall-related risk factors should be assessed in order to identify patients at highest immediate risk of subsequent NVF.

## Competing interests

The authors declare that they have no competing interests.

## Authors’ contributions

KH participated in the design of the study, performed the data acquisition and statistical analyses. TG, PG, BW participated in the design of the study, and interpretation of analyses and data. SH, JB, PW, PB participated in the design of the study and interpretation of data. All authors read and approved the final manuscript.

## Pre-publication history

The pre-publication history for this paper can be accessed here:

http://www.biomedcentral.com/1471-2474/14/121/prepub
